# Theoretical Investigation of Stiffness and Vibration Frequency Enhancement in Novel Membrane-Wrapped Lattice Beams

**DOI:** 10.3390/ma19061247

**Published:** 2026-03-21

**Authors:** Peiyao Xi, Hao Zhou, Canghai Tan, Chuang Shi, Rongqiang Liu, Jianzhong Yang

**Affiliations:** 1 School of Mechatronics Engineering, Harbin Institute of Technology, Harbin 150001, China; peiyao.xi@outlook.com (P.X.); liurq@hit.edu.cn (R.L.); 2 Beijing Institute of Spacecraft System Engineering, China Academy of Space Technology, Beijing 100094, China; zhouhao2010@pku.edu.cn (H.Z.); jzyang1234@sina.com (J.Y.)

**Keywords:** membrane-wrapped lattice, additive manufacture, stiffness, three-point bending, natural frequency

## Abstract

Bending-dominated lattice structures offer superior stability but suffer from low stiffness and natural frequencies, posing resonance risks in aerospace applications. To address this, a novel Membrane-Wrapped Lattice (MWL) encapsulated by a micrometer-scale metallic film is proposed. A theoretical framework based on the tension-compression asymmetry of the membrane is established to analyze the influence of membrane thickness on the neutral axis shift, ultimately deriving analytical formulations for flexural stiffness and natural frequencies. MWL specimens with varying membrane thicknesses (0–50 μm) were fabricated via selective laser melting and adhesive bonding, then subjected to three-point bending and vibration tests. Results demonstrate that wrapping with a 50 μm 316 L stainless steel membrane increases the flexural stiffness by 128% and the fundamental natural frequency by 85%. The experimental measurements align well with theoretical and numerical predictions, validating this lightweight, high-stiffness design strategy.

## 1. Introduction

Lattice structures are highly customizable, lightweight porous materials and have garnered significant research attention due to their superior mechanical properties, such as high rigidity [1], high strength [2], high energy absorption capacity [3,4] and highly customizable features [5,6]. They are widely employed in applications ranging from load-bearing [7,8] and energy absorption buffers [9,10] to vibration isolation [11,12] across the satellite [13,14], aircraft [15,16], automotive [17], biomedical [18,19], and sectors [20,21].

Deshpande et al. [22] classified truss lattices into bending-dominated and stretching-dominated types based on nodal connectivity. Stretching-dominated topologies (such as pyramid [23] and Octet [24]) provide high stiffness but limited stability. Conversely, bending-dominated lattices, such as Body-Centered Cubic (BCC) [25] and Face-Centered Cubic (FCC) [26] exhibit stable deformation under large strains. While ideal for energy absorption, their inherently low stiffness limits their utility as primary load-bearing components, often leading to vibration-induced instabilities.

From a theoretical perspective, the mechanical modeling of lattice structures is typically established by analyzing their characteristic deformation modes. Understanding the underlying deformation mechanisms—whether stretching-dominated or bending-dominated—is essential for predicting macroscopic mechanical behavior. Wang et al. [27,28] conducted a comprehensive analysis on multi-layer modified FCC lattice structures to characterize their mechanical response. Similarly, Wei et al. [29,30] investigated the Kagome lattice structure, establishing a mechanical model based on its specific topological configuration and deformation behavior.

In aerospace applications, bending-dominated lattice structures are frequently adopted because of their excellent deformation stability. However, owing to their relatively low stiffness, bending-dominated lattices exhibit low natural frequencies, similar to those of rocket structures, which makes them susceptible to vibration-induced failure during launch.

Enhancing stiffness while maintaining minimal weight addition therefore remains a critical challenge. Various geometric strategies have been investigated to overcome this limitation. For instance, Zhang et al. [31] developed gradient lattices via nodal displacement to simultaneously improve stiffness and energy absorption. Zhao et al. [32] proposed reinforcing lattice joints to enhance elastic modulus and yield strength, while Wang et al. [33] introduced hierarchical struts to boost the mechanical performance of FCC lattices. Ni et al. [34] proposed a soft-skin-wrapped rigid tube structure to enhance energy absorption by guiding the deformation mode.

In this study, a novel membrane-wrapped lattice (MWL) structure is proposed, in which a micrometer-scale metallic film is conformally attached to the outer surface of the lattice core. From a mechanical perspective, the film acts as a tension-only element, exhibiting negligible resistance to compression and bending. While it effectively constrains structural deformation under tensile loading, its lack of compressive and bending stiffness ensures that the intrinsic deformation mode of the lattice core remains unchanged. Accordingly, the mechanical behavior of the metallic film is described using membrane theory, enabling an analytical investigation of the flexural stiffness and natural vibration characteristics of the proposed MWL structure.

## 2. Materials and Methods

### 2.1. Structural Design

The membrane-wrapped lattice (MWL) structure is a hybrid architecture composed of an inner lattice core and a conformal outer membrane. Specifically, it is constructed by wrapping a micrometer-scale metallic film around the exterior of a porous lattice structure. Acting as a tension-only element with negligible bending or compressive capacity, the membrane restricts deformation specifically in the tensile region. Leveraging its high stiffness, this design significantly enhances the mechanical performance without altering the inherent deformation mode.

**Figure 1:**
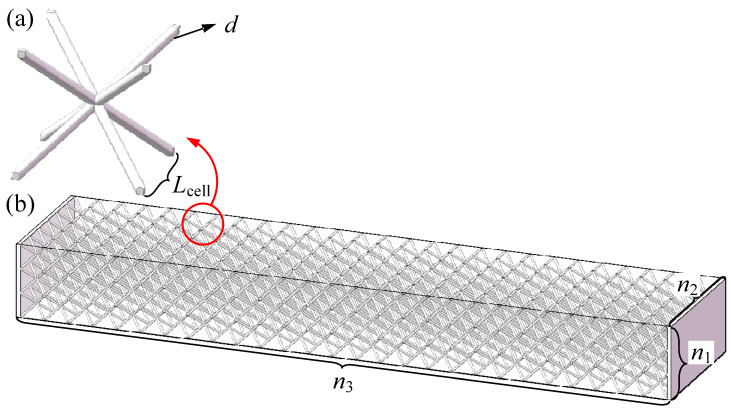
Structural design of (**a**) lattice cell and (**b**) membrane-wrapped lattice beam.

In this study, the Body-Centered Cubic (BCC) topology is selected as the representative lattice unit cell. As illustrated in Figure 1a, the unit cell has a side length of *L*_cell_ and consists of eight identical circular struts of diameter *d* intersecting at a central node. The lattice core is formed by tessellating these BCC unit cells along three orthogonal axes, as shown in Figure 1b. The numbers of unit cells in the width, height, and length directions are denoted by *n*_1_, *n*_2_, and *n*_3_, respectively. The width and height of the core is b and h. Finally, a metallic membrane with thickness *c* is wrapped around, fully enclosing the core, resulting in the complete MWL structure.

### 2.2. Fabrication of Specimens

**Figure 2:**
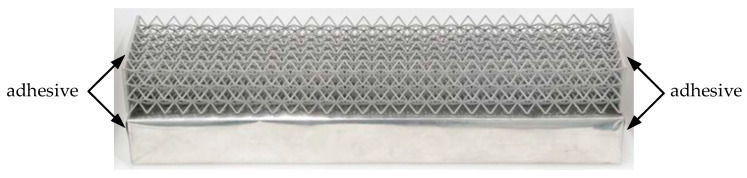
Unwrapped and membrane-wrapped lattice beam.

The specimens were fabricated from 316 L stainless steel using selective laser melting by BLT-S400 manufacturing system (BLT, Xi’an, China), as shown in Figure 2. The BCC unit cell size was 12.5 mm, with a strut diameter of 1.27 mm (l/d = 8.5). The specimens comprised 3 × 6 × 27 unit cells, resulting in overall dimensions of 37.5 mm × 75 mm × 314.5 mm. A 316 stainless steel membrane was wrapped around the core, covering the top, bottom, and front and rear surfaces to fully enclose the lattice structure. The membrane was bonded to the core along the two lateral sides using 3M Scotch-Weld DP420 (3M company, Saint Paul, MN, USA) adhesive to ensure firm attachment. After wrapping with membranes of 10 μm, 30 μm, and 50 μm thickness, the overall mass increased by approximately 1%, 2%, and 3%, respectively. The increased mass is sufficiently small to justify neglecting the mass change in the subsequent analysis.

### 2.3. Three-Point Bending Tests and Finite Element Method Simulation

**Figure 3:**
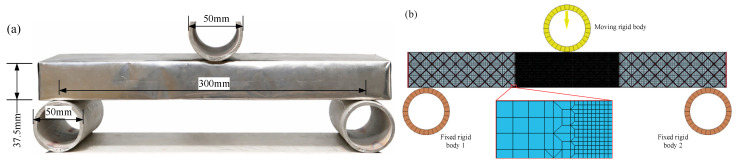
Configuration of three-point bending (**a**) tests, and (**b**) FEM.

As shown in Figure 3a, three-point bending tests were performed on a Instron 5969 testing machine (Instron, Norwood, MA, USA) under displacement control of 2 mm/min. The bending span was set to 300 mm. The applied load and corresponding displacement were continuously recorded throughout the testing machine.

Finite element method (FEM) simulations were conducted using LS-DYNA R11.0 to investigate the stress distribution of the MWL structures, as shown in Figure 3b. The numerical model was constructed to faithfully replicate the experimental three-point bending configuration, with all geometric dimensions identical to those used in the experiments. In the numerical simulation, the membrane was modeled using fully integrated shell elements with five integration points through the thickness. To improve numerical accuracy, the structures were discretized using three-dimensional solid elements, and mesh refinement was applied in the central region of the membrane. The supporting and loading rollers were modeled as rigid bodies to replicate the experimental setup. The two lower rollers were fixed in all translational degrees of freedom, while the upper roller was prescribed with a vertical displacement to simulate displacement-controlled loading. The material properties were obtained from tensile tests, as listed in Table 1.

### 2.4. Vibration Tests

**Figure 4:**
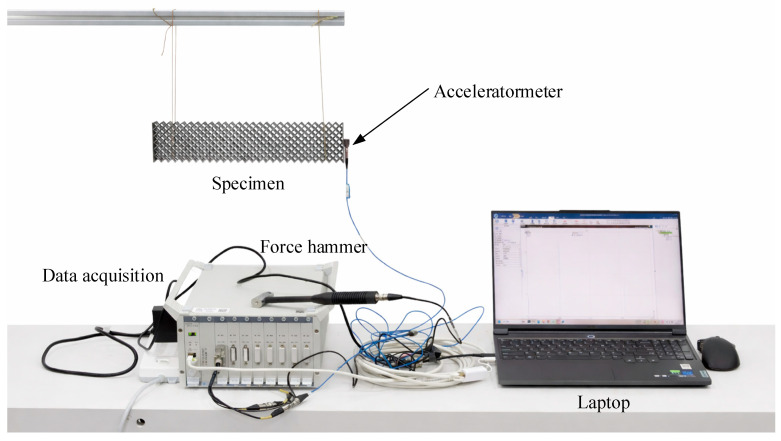
Configuration of vibration test.

To characterize the natural frequency characteristics of the MWL structure, vibration tests were conducted as shown in Figure 4. The specimen was suspended using a soft suspension method to approximate free–free boundary conditions, thereby minimizing the influence of external constraints on the measured dynamic response. The beam was excited using an instrumented impact hammer, and the acceleration response was measured by a lightweight accelerometer mounted at the free end of the beam. The mass of the accelerometer was less than 2% of the beam mass to avoid significant mass-loading effects. The excitation force and acceleration response signals were sampled at a frequency of 5 kHz using a Donghua dynamic signal acquisition and analysis system. Each test was repeated three times, and the frequency response functions (FRFs) were obtained through FFT-based processing of the measured signals. The fundamental natural frequencies were identified using the peak-picking method from the averaged FRFs.

## 3. Theoretical Analyses

### 3.1. Mechanical Properties of the Lattice Core

In this section, a theoretical approach for predicting bending stiffness of membrane- wrapped lattice structures subjected to bending deformation is raised. Generally, the periodic lattice core can be homogenized as an equivalent orthotropic material, and the constitutive relationship between stress and strain is described by the compliance matrix **S** [35]:

(1) $$[S]=\left[\begin{array}{ccc} \frac{1}{E_{11}} & -\frac{\nu_{21}}{E_{22}} & -\frac{\nu_{31}}{E_{33}}\\ -\frac{\nu_{12}}{E_{11}} & \frac{1}{E_{22}} & -\frac{\nu_{32}}{E_{33}}\\ -\frac{\nu_{13}}{E_{11}} & -\frac{\nu_{23}}{E_{22}} & \frac{1}{E_{33}} \end{array}\right]$$

For a slender beam with a high aspect ratio, the influence of shear deformation is negligible compared to the bending deformation. Under this assumption, the internal stress state of the beam is dominated by longitudinal tension and compression induced by the bending moment. Consequently, the effective elastic modulus of the lattice core is characterized by the longitudinal elastic modulus:

(2) $$E_{core}=E_{11}$$

### 3.2. Theoretical Analysis of Bending Stiffness of MWL

In this section, a theoretical approach for predicting the bending stiffness of MWL beam, *D*_MWL_, is introduced. And the following assumptions are adopted during the theoretical analysis:

(1) Due to the extremely small thickness of the membrane, its yielding force is correspondingly low. As a result, the membrane can be reasonably assumed to be incapable of sustaining compressive stresses, and its contribution under compression may therefore be neglected.
(2) The overall dimensions and the relative density of the structure are assumed to remain unchanged after membrane wrapping.
(3) The cross-sectional dimensions of the MWL beam remain unchanged after deformation.
(4) The constituent materials are assumed to be linearly elastic, and the plane section assumption is adopted, implying that plane sections before bending remain plane after bending.

**Figure 5:**
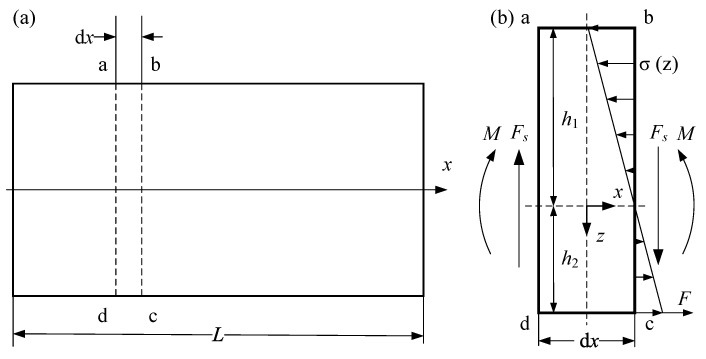
Theoretical model and sectional analysis of the MWL beam: (**a**) Model of MWL beam and (**b**) force analysis of an infinitesimal beam element.

A differential beam element of length dx is considered, as shown in Figure 5a. After bending deformation, the element exhibits a curvature *κ*. The axial strain distribution over the cross-section is:

(3) ε(z)=κz

where *z* is the distance from the neutral axis.

For a conventional beam with symmetric tensile and compressive behavior, the neutral axis coincides with the geometric midline. However, in the MWL beam, the tension–compression asymmetry introduced by the membrane leads to a downward shift of the neutral axis from the geometric midline, as shown in Figure 5b [36]. Consider an infinitesimal beam element of length d*x* subjected to a bending moment M and a shear force Fs. Under bending, a normal stress distribution *σ*_z_ is generated across the cross-section. Let the distances from the neutral axis to the upper and lower surfaces be *h*_1_ and *h*_2_, respectively, satisfying:

(4) $$h=h_{1}+h_{2}$$

Next, a force analysis is performed. Equilibrium of internal forces on the cross-section requires that the compressive force equals the tensile force:

(5) $$b\int_{-h_{1}}^{0}-\varepsilon (z)E_{core}dz=b\int_{0}^{h_{2}}\varepsilon (z)E_{core}dz+2c\int_{0}^{h_{2}}\varepsilon (z)E_{\mathrm{membrane}}dz+F$$

where *b* denotes the cross-sectional width, *c* represents the membrane thickness, and *F* denotes the tensile force carried by the outermost membrane layer, given by:

(6) $$F=\kappa h_{2}bcE_{\mathrm{membrane}}$$

Accordingly, by substituting Equation (4) into Equation (5), the position of the neutral axis can be obtained as:

(7) $$h_{1}=\sqrt{{h_{2}}^{2}+\frac{2\left(cE_{\mathrm{membrane}}{h_{2}}^{2}+bcE_{\mathrm{membrane}}h_{2}\right)}{bE_{core}}}$$

Next, the bending stiffness is evaluated. The total bending moment can be expressed as the sum of the moments from the core and the tensile membrane:

(8) $$M_{MWL}=b\int_{-h_{1}}^{h_{2}}\varepsilon (z)E_{core}zdz+2c\int_{0}^{h_{2}}\varepsilon (z)E_{\mathrm{membrane}}zdz+bc\varepsilon (h_{2})E_{\mathrm{membrane}}h_{2}=D_{MWL}\kappa$$

By substituting Equation (3) into Equation (8), the bending stiffness of the MWL beam can be obtained as:

(9) $$D_{MWL}=\frac{bc{h_{2}}^{2}}{3}\left[\frac{\left({h_{1}}^{3}+{h_{2}}^{3}\right)}{c{h_{2}}^{2}}E_{core}+\frac{2h_{2}+3b}{b}E_{\mathrm{membrane}}\right]$$

Next, the bending stiffness of the MWL beam is compared with that of the lattice core to quantify the enhancement induced by membrane wrapping. A dimensionless parameter *χ*_D_ is introduced as the enhancement factor, defined as:

(10) $$\chi_{D}=\frac{D_{MWL}}{D_{core}}$$

where *D*_core_ denotes the bending stiffness of the bare lattice core, given by *D*_core_ = *E*_core_*bh*^3^/12.

By introducing geometric parameters *η* = *h*_1_/*h*_2_, *δ* = *c*/*h* and *ξ* = *h*/*b*, together with stiffness ratio *λ* = *E*_membrane_/*E*_core_, the expression for *χ* can be simplified as:

(11) $$\chi_{D}=4\left[1-2\eta +(1+\lambda \delta )\eta^{2}\right]$$

### 3.3. Theoretical Analysis of Vibration Behavior

In this section, the vibration performance of the MWL beam is further analyzed. For a slender Euler–Bernoulli beam, the governing equation is given by:

(12) $$D_{MWL}\frac{\partial^{4}w(x,t)}{\partial x^{4}}+\rho A\frac{\partial^{2}w(x,t)}{\partial t^{2}}=0$$

By letting:

(13) $$a=\sqrt{\frac{D_{MWL}}{\rho A}}$$

Equation (12) can be rewritten as:

(14) $$\frac{\partial^{4}w(x,t)}{\partial x^{4}}=-\frac{1}{a^{2}}\frac{\partial^{2}w(x,t)}{\partial t^{2}}$$

The general solution of this equation is:

(15) w=X(x)T(t)

where the function *X*(x) is expressed as:

(16) $$X(x)=C_{1}\cos\beta x+C_{2}\sin\beta x+C_{3}\cosh\beta x+C_{4}\sinh\beta x$$

The corresponding natural frequencies can then be determined by imposing different boundary conditions. However, as reported in previous studies [37], for all boundary conditions, the natural frequencies are proportional to the parameter a, i.e.,

(17) $$f_{i}\propto a\propto \sqrt{D_{MWL}}$$

Accordingly, following the definition introduced above, a frequency enhancement factor *χ*_f_ is defined. It can further be shown that the frequency enhancement factor *χ*_f_ is related to the stiffness enhancement factor *χ*_D_ as:

(18) $$\chi_{f}=\frac{f_{SWL}}{f_{core}}=\sqrt{\chi_{D}}$$

In practical applications, the beam slenderness ratio cannot always be guaranteed to exceed 5. For relatively short and thick beams, the Timoshenko beam theory becomes more appropriate, and the governing equation of vibration is given by:

(19) $$D_{MWL}\frac{\partial^{4}w}{\partial x^{4}}+\rho A\frac{\partial^{2}w}{\partial t^{2}}-\rho I\left(1+\frac{E}{k^{\prime }G_{MWL}}\right)\frac{\partial^{4}w}{\partial x^{2}\partial t^{2}}+\frac{\rho^{2}I}{k^{\prime }G_{MWL}}\frac{\partial^{4}w}{\partial t^{4}}=0$$

The solution can also be expressed in the form of Equation (15). For a simply supported Timoshenko beam, the natural frequency is given by:

(20) $$\omega_{i}=\sqrt{\frac{D_{MWL}}{\rho A}}\frac{i^{2}\pi^{2}}{L^{2}}\left[1-\frac{\pi^{2}i^{2}}{2L^{2}}\sqrt{\frac{I}{A}}\left(1+\frac{E}{k’G_{MWL}}\right)\right]$$

It can be observed that, the frequency enhancement of a Timoshenko beam is lower than the square-root scaling with bending stiffness predicted by the Euler–Bernoulli beam model, due to the inclusion of shear deformation and rotational inertia, that is:

(21) $$\chi_{f}=\frac{f_{SWL}}{f_{core}}<\sqrt{\chi_{D}}$$

**Figure 6:**
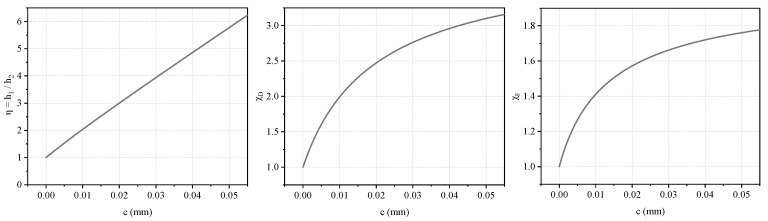
Variation of *η*, *χ*_D_, and *χ*_F_ with membrane thickness *c*.

From the above analysis, the variations of *η*, *χ*_D_, and *χ*_F_ with respect to the membrane thickness *c* can be obtained, as illustrated in Figure 6.

## 4. Results and Discussions

### 4.1. Results

#### 4.1.1. Bending Result

**Figure 7:**
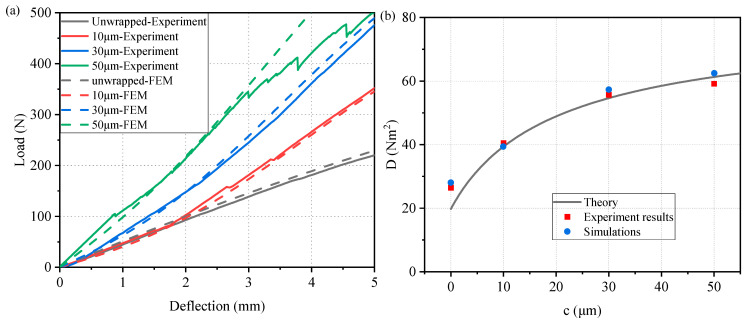
Comparison of theoretical, experimental, and FEM results for bending: (**a**) Load–deflection curve, (**b**) Bending stiffness–membrane thickness curve.

The flexural behavior of the MWL structures was experimentally evaluated using three-point bending tests on specimens with membrane thicknesses of 0, 10, 30, and 50 μm. Figure 7a presents the representative load–deflection curves obtained from the experiments and FEM. The experimental results exhibit good agreement with the FEM results, thereby validating the accuracy of the finite element model.

Figure 7b presents the flexural stiffness of the MWL as a function of the membrane thickness. The theoretical prediction is shown as the black continuous curve, while the experimental and FEM results are represented by discrete data points. As the membrane thickness increases, the flexural stiffness exhibits a clear monotonic increase. An agreement between the experimental measurements and the theoretical curve is observed over the entire range of membrane thicknesses, indicating that the proposed theoretical model accurately captures the stiffening effect induced by the membrane.

#### 4.1.2. Vibration Results

**Figure 8:**
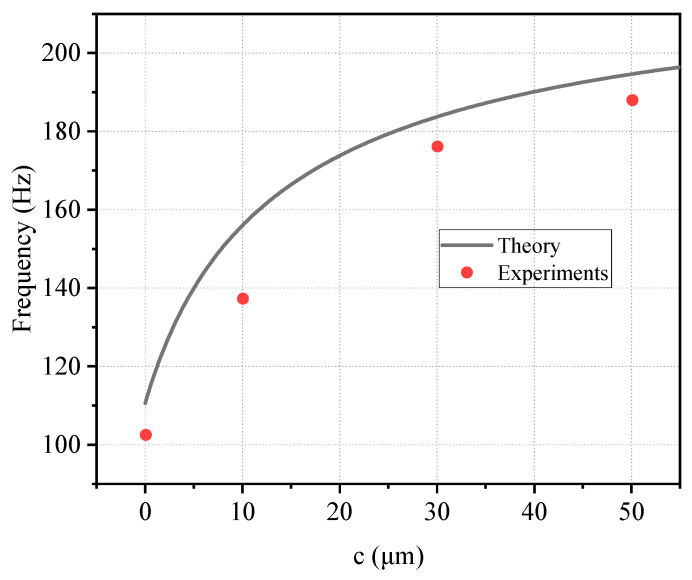
Comparison of theoretical and experimental results for vibration.

Figure 8 presents the fundamental natural frequencies obtained from both the theoretical model described in Section 3 and the experimental measurements for specimens with membrane thicknesses of 0, 10, 30, and 50 μm. A monotonic increase in natural frequency is observed with increasing membrane thickness. The experimental results exhibit excellent agreement with the theoretical predictions over the entire thickness range. The theoretical curve accurately captures the measured data points, thereby validating the proposed analytical framework. This consistent trend confirms that the high-stiffness membrane effectively enhances the global dynamic stiffness of the structure and demonstrates that the model properly accounts for the contribution of bending deformation to the dynamic response of the MWL beam.

### 4.2. Synergistic Mechanism of the MWL

**Figure 9:**
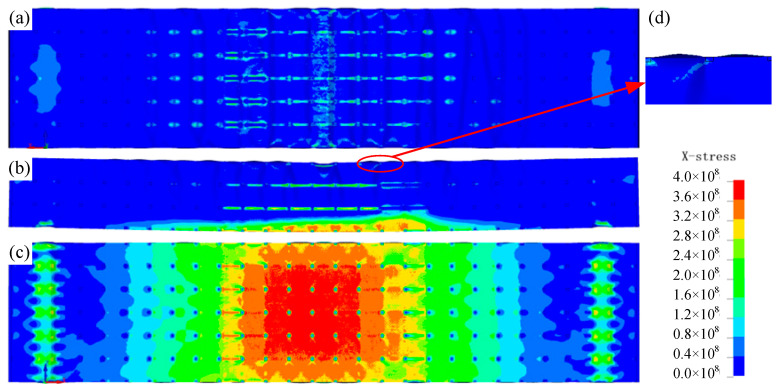
Stress distribution: (**a**) top view, (**b**) front view, (**c**) bottom view, (**d**) enlarged view of buckling on the top membrane.

Figure 9 illustrates the stress distribution obtained from the numerical simulations along the beam length when deflection is 3 mm. It can be observed from Figure 9a,c that tensile stress is concentrated at the mid-span of the bottom surface, whereas the top surface experiences negligible stress in this direction, confirming the validity of the adopted assumptions. Figure 9d illustrates the deformed shape of the upper membrane, where it is evident that buckling has already occurred. Moreover, as shown in Figure 9b, significant stress is present only within approximately the lower 1/4 of the cross-section, indicating a downward shift of the neutral axis.

This behavior arises from the tension-only load-carrying mechanism of the membrane, which ignores compressive stress on the upper surface while promoting tensile stress on the lower surface. It can be observed that the membrane in the central region is subjected to uniform tensile stress along the beam length. This stands in sharp contrast to the face sheets of traditional sandwich panels, which typically resist loads via localized bending. Compared to the sheets in a sandwich panel structure, the membrane increases the length of the deformation zone, thereby significantly maximizing structure utilization.

The experimental and theoretical results confirm that the flexural stiffness of the MWL structure increases monotonically with membrane thickness, validating the proposed design strategy. As derived in Section 3.2, the wrapping membrane shifts the neutral axis away from the geometric center towards the tension side, leading to an asymmetric stress distribution across the section. Consequently, the tensile stress is predominantly borne by the bottom membrane, while the lattice core sustains the shear loads. This decoupling of stress components allows the MWL structure to effectively overcome the low-stiffness drawback of bending-dominated lattices.

## 5. Conclusions

In this study, a novel Membrane-Wrapped Lattice (MWL) structure was proposed to address the low-stiffness and low-frequency drawbacks of conventional bending-dominated lattice materials. The key conclusions are summarized as follows:

(1) The introduction of a tension-only membrane induces pronounced tension–compression asymmetry in the MWL beam, leading to a downward shift of the neutral axis and an asymmetric stress distribution across the cross-section. Based on this mechanism, analytical formulations for flexural stiffness and natural frequencies were derived.
(2) Membrane wrapping significantly enhances structural performance. A 50 μm-thick 316 L stainless steel membrane increases the flexural stiffness by 128% and the fundamental natural frequency by 85% compared with the unwrapped lattice, while introducing only about a 1% mass increase.
(3) The theoretical predictions were rigorously validated through experimental testing, and the underlying assumptions were verified via numerical simulations. Finite element analysis confirmed that the internal stress distribution aligns with the theoretical model, specifically validating the efficacy of the tension-dominant load-carrying mechanism in enhancing global structural rigidity.

## Figures and Tables

**Table 1:** Material properties in FEM analysis.

	Elastic Modulus (GPa)	Poisson’s Ratio	Yield Strength (MPa)	Ultimate Tensile Strength (MPa)	Fracture Elongation
SLM-316 L (lattice core)	11.1	0.3	564.5	860.3	0.36
316 L (membrane)	12.4	0.3	342.3	975.2	0.43

## Data Availability

The original contributions presented in this study are included in the article. Further inquiries can be directed to the corresponding authors.

## References

[1] Yu X., Zhou J., Liang H., Jiang Z., Wu L. (2018). Mechanical metamaterials associated with stiffness, rigidity and compressibility: A brief review. Prog. Mater. Sci..

[2] Inoma A., Ibhadode O. (2025). A Novel Dual Curved Cubic (DCC) structure with improved compressive strength. Int. J. Mech. Sci..

[3] Kibaroglu D., Katti A., Drebenstedt C., Hipke T., Krupp U., Haase C. (2025). Enhancing energy absorption performance in additively manufactured lattice structures: A synergistic approach to geometry and alloy design. Mater. Des..

[4] Wu X., Guo H., Zhang J. (2025). Bi-Surface Induction in Biomimetic Multi-Gradient Foam-Filled Tubes With Enhanced Energy Absorption: Theory, Experiment, and Simulation. ASME J. Appl. Mech..

[5] Lin C., Zhang L., Liu Y., Liu L., Leng J. (2020). 4D printing of personalized shape memory polymer vascular stents with negative Poisson’s ratio structure: A preliminary study. Sci. China Technol. Sci..

[6] Guo H., Zhang J. (2024). Performance-Oriented and Deformation-Constrained Dual-topology Metamaterial with High-Stress Uniformity and Extraordinary Plastic Property. Adv. Mater..

[7] Chen X., Ji Q., Iglesias Martínez J.A., Tan H., Ulliac G., Laude V., Kadic M. (2022). Closed tubular mechanical metamaterial as lightweight load-bearing structure and energy absorber. J. Mech. Phys. Solids.

[8] Guo Z., Yang F., Li P., Li L., Zhao M., Shi J., Zhang L., Cai Y. (2024). A partially hollow BCC lattice structure with capsule-shaped cavities for enhancing load-bearing and energy absorption properties. Eng. Struct..

[9] Li B., Liu H., Zhang Q., Chai C., Wang J., Yang J., Yang X. (2024). Crashworthiness and stiffness improvement of a variable cross-section hollow BCC lattice reinforced with metal strips. Aerosp. Sci. Technol..

[10] Zhao M., Cui J., Chen L., Jin K., Zeng Z. (2025). Enhanced mechanical properties and energy absorption of lattice metamaterials inspired by crystal imperfections. Compos. Struct..

[11] An X., Yuan X., Sun G., Hou X., Fan H. (2024). Design of lattice cylindrical shell meta-structures for broadband vibration reduction and high load-bearing capacity. Thin-Walled Struct..

[12] Wang Y., Chen X., Sun Y., Zhang J., Hu J., Bai L. (2024). Full-band vibration isolation and energy absorption via cuttlebone-inspired lattice structures. Int. J. Mech. Sci..

[13] Zhang X., Zhou H., Shi W., Zeng F., Zeng H., Chen G. (2018). Vibration Tests of 3D Printed Satellite Structure Made of Lattice Sandwich Panels. AIAA J..

[14] Boschetto A., Bottini L., Macera L., Vatanparast S. (2023). Additive Manufacturing for Lightweighting Satellite Platform. Appl. Sci..

[15] Wang T., An J., He H., Wen X., Xi X. (2021). A novel 3D impact energy absorption structure with negative Poisson’s ratio and its application in aircraft crashworthiness. Compos. Struct..

[16] Kilimtzidis S., Kotzakolios A., Kostopoulos V. (2023). Efficient structural optimisation of composite materials aircraft wings. Compos. Struct..

[17] Hou W., He P., Yang Y., Sang L. (2023). Crashworthiness optimization of crash box with 3D-printed lattice structures. Int. J. Mech. Sci..

[18] Vaiani L., Uva A.E., Boccaccio A. (2023). Structural and topological design of conformal bilayered scaffolds for bone tissue engineering. Thin-Walled Struct..

[19] Tseng S.-F., Wang I.-H., Chang C.-M., Lee C.-C., Yeh D.-Y., Chen T.-W., Yeh A.-C. (2022). Mechanical characteristic comparison of additively manufactured Ti–6Al–4V lattice structures in biocompatible bone tissue growth. Mater. Sci. Eng. A.

[20] Bang J., Chun B., Lim J., Han Y., So H. (2023). Ultra-Broad Linear Range and Sensitive Flexible Piezoresistive Sensor Using Reversed Lattice Structure for Wearable Electronics. ACS Appl. Mater. Interfaces.

[21] Wu P., Yu T., Zhao L., Chen M. (2024). Magnetic field-assisted 3D printing of magnetic self-powered sensors. Virtual Phys. Prototyp..

[22] Deshpande V.S., Ashby M.F., Fleck N.A. (2001). Foam topology: Bending versus stretching dominated architectures. Acta Mater..

[23] Queheillalt D.T., Wadley H.N.G. (2005). Pyramidal lattice truss structures with hollow trusses. Mater. Sci. Eng. A.

[24] Mishra A.K., Kumar A. (2023). Performance of asymmetric octet lattice structures under compressive and bending loads. Eng. Fail. Anal..

[25] Ushijima K., Cantwell W.J., Mines R.A.W., Tsopanos S., Smith M. (2010). An investigation into the compressive properties of stainless steel micro-lattice structures. J. Sandw. Struct. Mater..

[26] Wang P., Bian Y., Yang F., Fan H., Zheng B. (2020). Mechanical properties and energy absorption of FCC lattice structures with different orientation angles. Acta Mech..

[27] Wang P., Yang F., Li P., Zheng B., Fan H. (2021). Design and additive manufacturing of a modified face-centered cubic lattice with enhanced energy absorption capability. Extrem. Mech. Lett..

[28] Wang P., Yang F., Ru D., Zheng B., Fan H. (2021). Additive-manufactured hierarchical multi-circular lattice structures for energy absorption application. Mater. Des..

[29] Wei K., Yang Q., Ling B., Xie H., Qu Z., Fang D. (2018). Mechanical responses of titanium 3D kagome lattice structure manufactured by selective laser melting. Extrem. Mech. Lett..

[30] Wei K., Yang Q., Yang X., Tao Y., Xie H., Qu Z., Fang D. (2020). Mechanical analysis and modeling of metallic lattice sandwich additively fabricated by selective laser melting. Thin-Walled Struct..

[31] Zhang S., Yang F., Li P., Bian Y., Zhao J., Fan H. (2022). A topologically gradient body centered lattice design with enhanced stiffness and energy absorption properties. Eng. Struct..

[32] Zhao M., Liu F., Fu G., Zhang D.Z., Zhang T., Zhou H. (2018). Improved Mechanical Properties and Energy Absorption of BCC Lattice Structures with Triply Periodic Minimal Surfaces Fabricated by SLM. Mater..

[33] Wang P., Yang F., Zheng B., Li P., Wang R., Li Y., Fan H., Li X. (2023). Breaking the Tradeoffs between Different Mechanical Properties in Bioinspired Hierarchical Lattice Metamaterials. Adv. Funct. Mater..

[34] Ni H., Zhu S., Chen L., Liu T., Hou X. (2025). Soft-rigid composite metamaterials: Reusable energy absorption via radial sinusoidal interfaces and frictional dissipation. Eng. Struct..

[35] Jones R.M. (1999). Mechanics of Composite Materials.

[36] Yao W.-J., Ye Z.-M. (2004). Analytical solution of bending-compression column using different tension-compression modulus. Appl. Math. Mech..

[37] Han S.M., Benaroya H., Wei T. (1999). Dynamics of transversely vibrating beams using four engineering theories. J. Sound Vib..

